# Putting Coins in the Mouth

**Published:** 1880-01

**Authors:** 


Putting Coins in the Mouth.—We often see persons put a piece of money between the lips to hold it while their hands are occupied in putting on their gloves or adjusting the dress. It is not only untidy, but it is unsafe, because coins may thus convey the seeds of disease.

